# Predictive and Diagnostic Biomarkers of Anastomotic Leakage: A Precision Medicine Approach for Colorectal Cancer Patients

**DOI:** 10.3390/jpm11060471

**Published:** 2021-05-25

**Authors:** Mark Gray, Jamie R. K. Marland, Alan F. Murray, David J. Argyle, Mark A. Potter

**Affiliations:** 1 The Royal (Dick) School of Veterinary Studies and Roslin Institute, University of Edinburgh, Easter Bush, Roslin, Midlothian, Edinburgh EH25 9RG, UK; david.argyle@roslin.ed.ac.uk; 2 School of Engineering, Institute for Integrated Micro and Nano Systems, University of Edinburgh, Scottish Microelectronics Centre, King’s Buildings, Edinburgh EH9 3FF, UK; jamie.marland@ed.ac.uk; 3 School of Engineering, Institute for Bioengineering, University of Edinburgh, Faraday Building, The King’s Buildings, Edinburgh EH9 3DW, UK; alan.murray@ed.ac.uk; 4 Department of Surgery, Western General Hospital, Crewe Road, Edinburgh EH4 2XU, UK; mark.potter@ed.ac.uk

**Keywords:** colorectal cancer, intestinal anastomosis, anastomotic leak, biomarkers, precision medicine

## Abstract

Development of an anastomotic leak (AL) following intestinal surgery for the treatment of colorectal cancers is a life-threatening complication. Failure of the anastomosis to heal correctly can lead to contamination of the abdomen with intestinal contents and the development of peritonitis. The additional care that these patients require is associated with longer hospitalisation stays and increased economic costs. Patients also have higher morbidity and mortality rates and poorer oncological prognosis. Unfortunately, current practices for AL diagnosis are non-specific, which may delay diagnosis and have a negative impact on patient outcome. To overcome these issues, research is continuing to identify AL diagnostic or predictive biomarkers. In this review, we highlight promising candidate biomarkers including ischaemic metabolites, inflammatory markers and bacteria. Although research has focused on the use of blood or peritoneal fluid samples, we describe the use of implantable medical devices that have been designed to measure biomarkers in peri-anastomotic tissue. Biomarkers that can be used in conjunction with clinical status, routine haematological and biochemical analysis and imaging have the potential to help to deliver a precision medicine package that could significantly enhance a patient’s post-operative care and improve outcomes. Although no AL biomarker has yet been validated in large-scale clinical trials, there is confidence that personalised medicine, through biomarker analysis, could be realised for colorectal cancer intestinal resection and anastomosis patients in the years to come.

## 1. Introduction

Colorectal cancer is the fourth most commonly diagnosed cancer in the world, with ~1.8 million new cases and ~0.7 million cancer-related deaths occurring per year. The disease accounts for 10% of all newly diagnosed cancers, meaning it is a significant social and economic burden for many countries throughout the world [1]. In this review, we briefly discuss disease staging, colorectal cancer treatments, pathophysiology of normal intestinal healing and the consequences of abnormal intestinal healing. We then go on to describe in depth how this knowledge has led to the identification of diagnostic and predictive biomarkers of anastomotic leakage, which could be used to provide a precision medicine approach for managing colorectal cancer patients.

## 2. Colorectal Cancer Staging and Treatment

Before instigating treatment, patients undergo investigations to define the stage of the cancer. This is typically done using the tumour, node, metastasis (TNM) classification system (developed by the Union for Interventional Cancer Control) whereby data are collected from physical examinations, imaging and endoscopy. Pathological classification will be based on histopathology from biopsy samples typically obtained during endoscopy. Depending on disease stage, various treatment options are available; however, for curative intent strategies, surgery will be the treatment of choice. UK estimates indicate that 66% of colon cancer and 63% of rectal cancer patients will receive surgery as part of their primary care [2]. Surgery encompasses the excision of diseased intestinal segments containing the tumour (resection) with the subsequent re-joining of the disease-free intestinal ends (anastomosis). This intestinal resection and anastomosis procedure can be performed either with hand-placed sutures, automatic stapling devices or through robotically assisted techniques. Regardless of which technique is used, the procedure aims to re-establish luminal and mural intestinal continuity. Records from the Association of Coloproctology of Great Britain and Ireland (ACPGBI) have shown that, within Ireland and the UK, ~20,000 patients undergo a large bowel resection and anastomosis every year. The majority of these procedures are performed to treat colorectal cancers. Colorectal cancer patient outcomes have improved over the years through advances in peri-operative management, the use of neoadjuvant and adjuvant radiotherapy and chemotherapy and through modifications of the surgical procedure. These advancements have undoubtably contributed to the improved 5-year survival rate, which is now almost 60% [2]. Unfortunately, no matter how safe the surgical procedure is regarded to be, complications can still occur. One such life-threatening complication that typically occurs following failure of the anastomotic site to heal correctly is termed an anastomotic leak (AL).

## 3. Anastomotic Leaks

The exact definition of what an AL is continues to be debated in the literature. The UK Surgical Infection Study Group defined an AL as ‘a leak of luminal contents from a surgical join between 2 hollow viscera’ [3]. However, a subsequent review of 97 papers highlighted a lack of definition consistency between studies, with 56 different terms being identified [4]. The lack of standardised terminology creates problems when comparing results generated between different studies. A more recent attempt by the International Multispecialty Anastomotic Leak Global Improvement Exchange Group has re-defined an AL as ‘a defect of continuity localised at the surgical site of the anastomosis, which creates a communication between intra-luminal and extra-luminal compartments.’ Using this classification method, three grades of AL, increasing in severity from A to C, have been described. Whereas grade A can be left untreated, grade B requires medical management and grade C requires revision surgery [5,6]. 

Whatever the definition used, an AL is typically diagnosed 5–8 days post-surgery, although some case reports have demonstrated that a delayed presentation beyond 30 days is possible [7]. While AL can occur in up to 24% of patients undergoing distal rectal surgery, combined rates for surgery performed at any level of the intestinal tract are accepted to be ~6–7% [8,9]. The development of an AL not only results in increased morbidity [10,11,12] and 30-day mortality rates [13], but in cancer patients, it has also been associated with higher local recurrence rates and decreased long-term survival, but not with distant recurrence [8,14,15,16,17]. One large study involving 1984 colorectal cancer patients showed that 5-year cancer-specific survival was 57.4% in those that developed an AL compared with 72% that recovered uneventfully. The 5-year local recurrence rates were also increased from 1.9% to 4.7% in those that developed an AL [18]. Several explanations for these poorer survival times and increased local recurrence rates have been proposed. As viable cancer cells have been identified within the intestinal lumen and on staple/suture lines, it is possible that, following an AL, these cells could exfoliate to extra-luminal tissues. Implantation of these cells in the serosal surface of the intestine, peritoneum or pelvis could lead to the development of local recurrence [19,20,21,22,23,24,25]. The inflammatory response related to an AL has been proposed to stimulate tumour proliferation and evolution to distant metastasis [26,27,28,29,30], with elevated levels of inflammatory markers such as C-reactive protein (CRP) associated with higher recurrence rates and impaired disease-free survival [31,32]. Intra-abdominal bacterial infections have also been suggested to stimulate neoangiogenesis, which may increase the risk of disease recurrence [33]. 

Revision surgery will be required in ~85–95% of AL patients, with 50% of symptomatic AL cases requiring permanent stoma formation [34]. Complications such as multi-organ failure, pneumonia, renal and cardiac issues, localised/generalised sepsis, wound infections and surgical site dehiscence are also commonly encountered secondary to an AL [11]. The intensive care and revision surgery needed to manage these conditions, as well as the AL itself, increases hospitalisation periods [35,36] and total treatment costs [36,37,38,39,40,41]. If a patient develops an AL, then early diagnosis is essential to decrease mortality rates and achieve a positive outcome [7,42,43,44,45]. One study suggested that a 2.5-day delay in instigating AL-specific treatments increased mortality rates from 24 to 39% [13], while a further study identified that a 7.6% decrease in survival was associated with every hour of delay from septic shock onset to when antibiotics were administered [46].

## 4. Intestinal Healing

Research into anastomotic healing and AL development has been acknowledged as a priority by numerous healthcare providers, including the National Health Service, National Institute for Health Research, the ACPGBI and the Colorectal Therapies Healthcare Technology Co-operative. Following a resection and anastomosis, intestinal healing has been described to occur in four stages [47,48,49].

➢ Stage 1. Haemostasis. Occurring immediately after intestinal injury, this stage involves platelet and coagulation cascade activation.
➢ Stage 2. Inflammation. Occurring within 10 days after intestinal injury, this stage involves surgical site recruitment of lymphocytes, neutrophils and macrophages.
➢ Stage 3. Proliferation. Occurring from 5 to 21 days after intestinal injury, this stage involves intestinal re-epithelisation through fibroblast recruitment and endothelial cell proliferation.
➢ Stage 4. Remodelling. The final stage of intestinal healing occurs from 21 days after intestinal injury and continues for up to 1 year. Here, collagen deposition and tissue remodelling can restore intestinal integrity.

## 5. Anastomotic Leak Pathophysiology and Risk Factors

In contrast to the well-documented and characterised stages of uneventful intestinal healing, relatively little is known about AL pathophysiology. Studies, however, have identified several surgical and patient-related risk factors that can influence AL development (Table 1) [8,9].

### 5.1. Patient-Related Factors

Patient age as well as gender have been identified as AL risk factors, with men and patients of either sex >60 years old being at increased risk of AL. Although the exact reason for this is unknown, it is thought that the narrower male pelvis and androgenic hormonal effects on the intestinal microvascular blood supply may play roles in AL development in male patients [50,51,52,53,54,55]. Multiple studies have also shown that the American Society of Anaesthesiologists (ASA) fitness score is also an independent AL risk factor. Patient scores ≥III are associated with a 2.5-fold increased AL risk [55,56]. ASA scores are generated using multiple patient-specific factors including nutritional status and medical history, which have themselves been identified as independent AL risk factors.

Adequate nutrition is an important factor for intestinal healing as it contributes to collagen synthesis and immune responses. Various studies have shown that patients who are malnourished (including obese patients), have pre-operative weight loss [57,58,59], anaemia or low albumin levels are at increased risk of AL [60,61]. Neo-adjuvant, pre-operative chemo-radiotherapy has also been shown to be an independent risk factor for AL. Radiotherapy causes poor intestinal healing and increased fibrosis by damaging the local intestinal vascular system and impairing fibroblast function [51,62,63,64]. Pre-operative blood transfusions, advanced tumour stage and tumours >5 cm have also been identified as AL risk factors [56,65]. Currently, there is no consensus as to whether metabolic diseases, such as diabetes mellitus, increase AL risk through impaired wound healing [66,67]; however, patients with pre-existing renal disease, or those that smoke or drink alcohol excessively, have been identified as high-risk for AL development [67,68,69,70]. 

Although the intestinal microbiome plays an important role in the health, physiology and healing of the intestine [71], specific bacterial infections have been demonstrated to increase AL risk. One early study exhibited that rats inoculated with *Pseudomonas aeruginosa*, 24 h following gastrectomy and oesophagoduodenostomy, demonstrated higher AL rates compared with rats that were also inoculated with *Pseudomonas aeruginosa* but received peri-operative antibiotics (95% vs. 6%) [72]. A subsequent human clinical trial supported these pre-clinical results by showing that reduced AL incidence (10.6% vs. 2.9%) and mortality rates (10.6% vs. 4.9%) were achieved in gastrectomy and oesophagojejunostomy patients treated with peri-operative antibiotics [73]. The authors suggested that antibiotics may play a protective role against AL development. Although the mechanisms by which bacterial infections contribute to AL development are not fully understood, matrix metalloprotease (MMP) activation and collagenolytic substances produced by anastomotic site bacteria may play a role [74]. Using a pre-clinical rat model, one study demonstrated that antibiotics, with efficacy against *Enterococcus faecalis* (a bacterial strain with high collagen-degrading activity), placed topically at the colorectal anastomotic site, reduced AL incidence, whereas intravenous antibiotics failed to eliminate anastomotic site *Enterococcus faecalis* and reduce AL rates [75]. Following these results, MMP inhibitors have undergone investigations for their ability to prevent AL. One meta-analysis concluded that although anastomotic strength in animal models can be improved through MMP inhibitors, human clinical trials have yet to demonstrate a role in decreasing AL rates [76].

### 5.2. Surgery-Related Factors

A significant AL risk factor is the anatomical location of where the anastomosis is performed in the gastrointestinal tract [77]. One systematic review identified that the highest rate of AL occurred in coloanal and colorectal anastomoses (5–19%). This rate was significantly greater than that seen in enteroentero (1–2%), ileorectal (3–7%), ileocolic (1–4%) and colocolic (2–3%) anastomoses [78]. Multiple studies have also shown that anastomotic position in relation to the anal verge is important; cancer resections performed in the mid/low rectum [79] or <6 cm from the anal verge [80] have been associated with significantly higher AL rates. Patients that require an emergency resection and anastomosis at any level of the gastrointestinal tract are also at higher risk [55].

When considering the surgical procedure itself, studies have failed to show AL rate differences between hand-sewn or stapled anastomoses [81,82] or between open abdominal procedures or laparoscopic surgery [83,84,85]. Studies investigating the advantages of robotically performed colorectal anastomoses have failed to show AL rate differences compared with laparoscopic resections [86,87,88]. Conflicting results have been reported as to what extent surgical experience can influence AL rates. Whilst one study demonstrated that surgery performed by high-volume colorectal surgeons may reduce AL, another failed to demonstrate AL rate differences when surgeon experience was taken into account [89,90]. Multiple firings of the stapling device and surgical times >3 h have also been identified as AL risk factors [56,65].

Poor intestinal tissue oxygenation (partial pressure of O_2_ in tissue; ptO_2_) has also been suggested to contribute to AL development. Iatrogenic surgical disruption of the peri-anastomotic microvascular blood supply or tension at the anastomotic site can compromise intestinal tissue perfusion. If local blood supply is unable to meet intestinal O_2_ requirements, this situation can lead to peri-anastomotic ischaemia and necrosis [48,49,91,92]. Adequate ptO_2_ is also required for collagen production, with O_2_ levels <15-20 mmHg associated with compromised synthesis. As submucosal collagen is the predominant tissue layer for anchoring sutures/staples in the early stages of anastomotic healing, inadequate collagen production could contribute to AL incidence [93]. 

## 6. Diagnosis

As already mentioned, early AL diagnosis and subsequent management is essential to reduce patient morbidity and mortality. Unfortunately, early diagnosis can be extremely difficult as there are no pathognomonic signs which can be specifically attributed to an AL. Patients can initially be asymptomatic while non-specific clinical signs can range from abdominal pain, ileus, pyrexia and cardiorespiratory issues to acute organ failure and sepsis. These wide-ranging clinical symptoms can be difficult to distinguish from those caused by normal post-operative inflammatory and physiological responses [94]. Based on clinical assessments, one study demonstrated that 69% of AL patients had a delayed diagnosis, of which the majority of patients presented with only cardiovascular symptoms [95]. Clinical assessment, regardless of experience and training, is therefore regarded as an inadequate technique for identifying high-risk AL patients or for its early diagnosis [96].

As clinical signs cannot be relied upon for AL diagnosis, clinicians use a variety of blood tests assessing inflammatory markers such as CRP and leukocytes. Unfortunately, these markers are again non-specific, with raised levels commonly occurring secondary to various post-operative complications, including chest, urinary and surgical site infections. Rather than using individual markers, one study assessed leukocyte number, creatinine levels, CRP levels, core temperature, urine production and systemic inflammatory response syndrome components. This combined approach was able to reduce the delay in AL diagnosis from 4 to 1.5 days [13]. Scoring systems have also been designed to predict AL risk. One study generated a scoring system based upon data from 1060 patients who underwent an anterior resection. Using known AL risk factors (intra-operative haemorrhage, gender and level of anastomosis), this study classified patients into low- (0–1), intermediate- (2–3) and high-risk (4–5 score) cohorts with AL rates of 1.9%, 8% and 16.1%, respectively [97]. The Colon Leak Score, which incorporates surgical and patient-specific risk factors, has also been developed to predict AL risk [98]. As well as these predictive scoring systems, others have looked to diagnose AL. The Dutch Leakage Score and the Modified Dutch Leakage Score have, unlike the previously mentioned predictive scoring systems, undergone clinical validation. Using clinical and physiological data with laboratory results, the derived Dutch Leakage Score has been shown to have a sensitivity of 97%, specificity of 53.5%, positive predictive value (PPV) of 16.1% and negative predictive value (NPV) of 99.5% for AL diagnosis (depending on the score cut-off values used). Meanwhile, the much simpler Modified Dutch Leakage Score, again depending on the score cut-off, could still produce a sensitivity of 97%, specificity of 56.8%, PPV of 17.2% and NPP of 99.5% for AL diagnosis [13,99,100].

Current clinical practices for AL diagnosis rely on abdominal imaging (plain radiographs, computed tomography (CT) scans or water-soluble contrast enemas (WSCE)), in conjunction with clinical and biochemical evaluation. Although CT is perhaps the most commonly used imaging modality for AL diagnosis, studies have shown it to have variable sensitivity and specificity. One retrospective study reported that only 47% of CT scans performed within 72 h of a patient requiring repeat surgery were diagnostic for an AL [101]. CT and rectal contrast radiography have been shown to have comparable sensitivity (57–60%) and specificity (100%) rates for AL diagnosis, greater than those of using clinical assessments alone (50% sensitivity and 89% specificity). The authors of this study suggested that whilst these imaging techniques gave false negative results, both were equally good for AL diagnosis [102]. Another large study in the 1970s analysed data from almost 2000 anterior resection patients. From the results, the authors suggested that although contrast studies could provide an indication of leak severity, they offered no diagnostic advantage over sigmoidoscopy and/or digital rectal examination, especially in patients that received a low anastomosis [103]. WSCE have also been shown to have higher false positive rates compared with digital rectal examination (6.4% vs. 3.5%) [104]. As plain abdominal X-rays can identify disrupted staple lines, a further study suggested that WSCE may only be required when intact staple lines, identified on radiographs, occur in conjunction with unrelenting clinical signs [105]. Another study demonstrated that WSCE used in colorectal or left-sided colonic anastomoses had sensitivity and specificity values of 52.2% and 86.7%, respectively, leading the authors to suggest that the test had little impact on improving early patient morbidity [106]. A retrospective study using data from colorectal patients demonstrated that WSCE detected ~83% of leaks, whereas only ~15% were identified using CT. This difference was most apparent for distal ALs, leading the authors to conclude that WSCE may be more beneficial in evaluating low anastomoses [107]. Several studies have highlighted that CT-based AL diagnosis is challenging [107,108]. These studies have indicated that the only reliable CT sign of an AL was the presence of peri-anastomotic liquid and air; extravasated contrast material from the intestinal luminal into the abdomen was not always present. Similarities in CT data between patients with and without AL were also observed. These results indicated that CT interpretation requires an experienced radiologist, and that radiological interpretation should be performed with knowledge of clinical data.

As a result of these conflicting findings, there is still no definitive consensus on which imaging modality should be used for AL diagnosis. Furthermore, a reluctance by clinicians to perform multiple scans due to cost, logistics and patient radiation exposure, combined with inherent delays incurred from the time of imaging to the interpretation of results, can significantly hinder prompt AL diagnosis. As a result of these imaging-based limitations, researchers and clinicians are looking at novel AL predictive and diagnostic methods that could lead to a more refined, precision medicine approach to patient management (Table 2).

## 7. Precision Medicine, Prognostic, Predictive and Pharmacodynamic Biomarkers

Current post-operative management of intestinal resection and anastomotic patients is principally focused on improving global and local tissue perfusion [109]. Post-operative preservation of normovolaemia [110], normothermia [111], delivering supplemental O_2_ [112] and goal-directed intravenous fluid therapy [113,114] can all reduce morbidity and improve outcomes. A pig model has also shown that intravenous colloids can increase perfusion and ptO_2_ in normal and peri-anastomotic colonic tissue [115]. Although these generic peri-operative patient management strategies may help to promote anastomotic healing and decrease AL rates, patient outcomes are likely to be improved by clinicians adopting a precision or personalised peri-operative treatment strategy.

Precision and personalised medicine encompass the idea that optimal patient management requires patient- and/or disease-specific factors to be considered. Although similar, specific personalised and precision medicine definitions highlight key differences in their concepts; whereas personalised medicine considers individual patient genetics, patient beliefs, social background, preferences and attitudes, precision medicine emphasises the importance of data collection and analysis [116]. Although both concepts have subtle differences, the term ‘precision medicine’ has become more extensively used. This has principally been due to unease amongst clinicians thinking that patients might misinterpret ‘personalised medicine’ as a technique by which drugs are developed for specific individuals [117,118]. Perhaps the clearest definition of what precision medicine is came from The National Research Council in America, who described it as ‘the tailoring of medical treatment to the individual characteristics of each patient…to classify individuals into subpopulations that differ in their susceptibility to a particular disease or their response to a specific treatment. Preventative or therapeutic interventions can then be concentrated on those who will benefit, sparing expense and side effects for those who will not’ [118]. To achieve the aims of this precision medicine concept, research has largely focused on the use of patient- or disease-specific biomarkers.

A biomarker can be any measurable tissue or bodily fluid biological substance that represents normal or abnormal physiological processes or pathological conditions [119]. There are four classical biomarker categories [120,121,122]:

1. Diagnostic. Identifies the presence of disease;
2. Predictive. Indicates the likely benefit of a specific treatment;
3. Prognostic. Indicates patient outcome, irrespective of treatment;
4. Pharmacodynamic. Allows monitoring treatment effectiveness.

**Figure 1:**
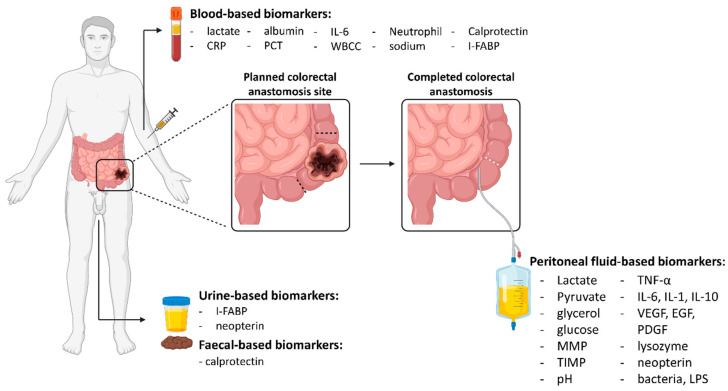
Patient samples used for biomarker assessment following a colorectal anastomosis in the treatment of colon cancer. (IL; interleukin, CRP; C-reactive protein, PCT; procalcitonin, WBCC; white blood cell count, I-FABP; intestinal fatty acid binding protein, MMP; matrix metalloproteinases, TIMP; tissue inhibitor of metalloproteinases, VEGF; vascular endothelial growth factor, EGF; epidermal growth factor, PDGF; platelet-derived growth factor, LPS; lipopolysaccharide). Figure created in Biorender.

A clinically useful biomarker is one that is obtained non-invasively, is easily assayed and provides results that have high sensitivity and specificity. Broadly speaking, for AL, these can be biomarkers of ischaemia, inflammation, tissue repair and the presence of bacterial contamination (Figure 1). All these potential biomarkers can be assessed through either blood or peritoneal fluid samples. These types of biomarkers have the potential to be assessed either intra-operatively, to predict which patients are at high risk of complications, or post-operatively, to identify which patients may require additional management to prevent an AL from developing or allow for its early diagnosis. In conjunction with clinical status, physiological parameters and imaging results, these types of biomarkers could be used to achieve a precision medicine approach for patients undergoing a resection and anastomosis.

## 8. Intra-Operative Techniques

During surgery, immediately following anastomosis, surgeons evaluate intestinal integrity through assessment of anastomotic doughnut completeness, air leak testing and/or endoscopic visualisation. Although air leak testing alone can reduce post-operative AL rates from 14% to 4% [123,124], intra-operative endoscopy can be performed with an air leak test and allows the surgeon to assess for vascular insufficiency, staple line bleeding, adequate tumour margins, iatrogenic intestinal injury and missed pathology [125,126,127]. One study demonstrated that routine intra-operative endoscopy identified anastomotic issues that required correcting in 10% of patients [125].

Further intra-operative assessment techniques have predominantly focused on intestinal ptO_2_ levels as a way of predicting which patients are at high risk of AL. Although surgeons evaluate macroscopic tissue appearance (colour, intestinal bleeding and palpable mesenteric pulses) as a surrogate for intestinal perfusion, these subjective techniques are unable to predict AL risk. To overcome this issue, various techniques have been developed to objectively measure intestinal tissue oxygen saturation (StO_2_) (visible light and near infrared spectroscopy) [128,129], tissue perfusion (laser fluorescence angiography, laser Doppler flowmetry) [130,131] and arterial haemoglobin O_2_ saturation (wireless handheld pulse oximeters) [132].

Visible light spectroscopy used in colorectal anastomoses has demonstrated that reduced tissue O_2_ saturation immediately after resection can predict AL. Interestingly, this study also showed that patients who recovered uneventfully demonstrated a significant intra-operative rise in StO_2_ in the proximal part of the anastomosis, which was not seen in those who developed an AL [128]. Animal studies have supported these results through comparing intestinal tissue oxygenation with staple size and by using wireless pulse oximeters [132,133]. In a recent human study, intra-operative colonic O_2_ saturation was measured with a pulse oximetry device placed on the colonic wall and evaluated for its ability to assess tissue viability and predict AL in colorectal anastomotic patients. The results showed that the risk of developing an AL was 4.2 times higher when post-anastomotic colonic StO_2_ was ≤90% of the pre-resection values. The authors suggested that low intra-operative colonic StO_2_ values were associated with AL occurrence [134]. Laser fluorescence angiography has also been shown, in a retrospective clinical trial of 402 patients, to reduce the number of patients that developed an AL. Out of the 22 patients that developed an AL, only seven (3.5%) were in the imaging group, compared with 15 (7.5%) in the control group [130]. Near infrared (NIR) fluorescent imaging has also been investigated for its intra-operative use as the energy range it uses is capable of penetrating deep into the intestinal walls and mesenteric tissues without causing thermal damage [135]. Coupled with indocyanine green, veins, arteries and capillaries can be identified, with vascular streams being used as an approximation of tissue perfusion. In animal models, this technique has been shown to predict the viability of ischaemic intestine [136] and an ongoing human clinical trial is assessing the use of NIR laparoscopy–indocyanine green to minimise leak occurrence compared with conventional white-light laparoscopy [137].

Clark O_2_ electrodes have also been investigated for their ability to measure intra-operative intestinal ptO_2_. In pre-clinical animal models, gradual intestinal perfusion reduction through sequential accurate intestinal artery ligation demonstrated that intestinal ptO_2_ measured before performing an anastomosis could predict AL occurrence [93,138,139]. In humans, Clark O_2_ electrodes have been used to provide intra-operative ptO_2_ reference values for the majority of the gastrointestinal tract [140]. Clinical trials have also shown that colonic ptO_2_ of less than either 20 mmHg, 50% of pre-resection values, 15% of arterial oxygen partial pressure (PaO_2_) or 40% of ptO_2_ at a control site were all associated with AL development. This study provided evidence that Clark O_2_ electrodes could be used to measure peri-anastomotic colonic ptO_2_ before, during and immediately after performing a resection and anastomosis and that, using defined cut-off values, the risk of developing an AL could be predicted [93].

Although these intra-operative techniques are well established, there are no standard guidelines as to which should be used. As a result of this, there is considerable variation between surgeons and hospitals [141]. To begin to address this, the European Society of Coloproctology Safe-anastomosis Programme in Colorectal Surgery (EAGLE) has been set up. Launched in 2019, this is an international, multicentre, cluster randomised controlled sequence study. EAGLE aims to recruit at least 2000 surgeons from 300 hospitals in order to collect data from >4500 patients who have undergone a right colectomy and ileocecal resection. The study results will be used as a quality improvement programme aimed at pre-operative risk stratification and standardising surgical techniques used for these patients [142].

## 9. Post-Operative Techniques

Many pre-clinical and clinical research studies have provided clear evidence that intra-operative assessment of anastomotic integrity and peri-anastomotic tissue perfusion can predict AL risk. Unfortunately, these techniques ultimately fail to encompass a precision medicine approach to patient care as they cannot be used to assess intestinal healing in the post-operative period. To overcome this, researchers are now looking at biomarkers which can be used post-operatively that allow continual patient monitoring and assessment of intestinal healing. These techniques have the goal of identifying high-risk patients and provide a means of early AL diagnosis. 

## 10. Biomarkers of Ischaemia

Under aerobic conditions, adenosine triphosphate (ATP) is efficiently generated during the conversion of glucose to pyruvate through glycolysis and the Krebs cycle. However, when O_2_ and glucose supply are limited and unable to meet the metabolic demands of a tissue, cells have to rely on anaerobic metabolism. Here, ATP is generated less efficiently from pyruvate being converted into lactate, with CO_2_ being released in the process. Ischaemic tissue microenvironments are therefore typically regarded as having low glucose and pyruvate levels in the presence of high lactate concentrations. Accumulated amounts of CO_2_ will also cause a reduction in tissue pH. If ischaemic conditions are prolonged, cells become damaged and, with the breakdown of their cell membranes, phospholipids are released, generating glycerol and free fatty acids. Although these individual ischaemic biomarkers can be measured, calculating lactate/pyruvate ratios (LP ratio) can characterise the aerobic/anaerobic metabolic balance, with higher values signifying ischaemia. Unfortunately, assessment of ischaemic biomarkers in blood has been shown to lack specificity for AL diagnosis [143]. To address this, ischaemic biomarker levels in peritoneal fluid have been investigated. Animal studies using microdialysis catheter fluid (small probes with dialysis membranes inserted into/onto the intestine) have shown that lactate, glucose and glycerol levels change with the metabolic alterations that occur in hypoxic [144] and ischaemia [145] conditions. Most human clinical studies have now focused on assaying ischaemic biomarkers in microdialysis catheter fluid or peritoneal fluid from abdominal drains. In a pilot study of eight patients undergoing right hemicolectomy, metabolic changes consistent with visceral ischaemia were identified in microdialysis catheter fluid several hours before clinical signs of AL became apparent [146]. A subsequent study characterised microdialysis catheter fluid reference ranges for the first 45 h following surgery in patients that recovered uneventfully from a variety of elective gastrointestinal operations [147].

### 10.1. Lactate/Pyruvate Ratio

High peritoneal LP ratios have been associated with AL in multiple clinical studies. In one study, patients undergoing anterior rectal resections had their LP ratio and glucose levels assessed for the first 6 days following surgery. Results indicated that the LP ratio in patients that developed an AL was significantly higher on days 5 and 6 following surgery. Unfortunately, due to low patient numbers, LP ratio cut-off values for predicting an AL could not be determined [148]. A further study using microdialysis catheters in 45 low anterior resection patients obtained fluid samples from the anastomotic site every 4 h. Lactate and LP ratios were found to be significantly raised in the four patients that developed an AL. Interestingly, in three patients who developed an AL >10 days following surgery, raised lactate and LP ratios were detected several days before clinical symptoms developed. Lactate levels in the remaining AL patient increased 18 h before clinical signs, with LP ratios only becoming elevated once clinical symptoms became evident [149]. A similar study again using microdialysis catheters obtained peritoneal fluid samples from patients every 2 h for the first 2 days, then every 4 h until 7 days following colorectal surgery. Higher peritoneal lactate and LP ratios and lower glycerol levels were seen immediately following surgery in patients that went on to develop an AL. These levels became significantly raised by the 4th day following surgery [150]. In another study that contained colorectal anastomoses, abdominal aortic aneurysm repairs, gastric procedures and cholecystectomy, results showed that increased peritoneal LP ratios and decreased glycerol levels were associated with ‘major intra-abdominal complications’ [151]. A further study involving 88 patients who underwent various abdominal procedures, including an intestinal resection and anastomosis, had their post-operative peritoneal and serum lactate levels assessed. Patients that had peritoneal/serum lactate level >4.5 or peritoneal lactate level >9.1 mM in the presence of pyrexia (>38.3 °C), raised white cell count (>12 × 10^9^/L), delayed passage of flatus (>72 h) and abdominal pain by the 4th day post-surgery were significantly associated with post-operative complications that required revision surgery (AL were included in this group) [152]. In slight contrast to these results, a further study which measured lactate, pyruvate, glycerol and glucose levels every 4 h for 5 days after patients underwent a left-sided colorectal anastomosis demonstrated that, in the three AL patients, lactate levels but not LP ratios were significantly elevated. Interestingly, in all the patients which developed an AL, the raised lactate levels occurred in the first 3 days following surgery [153].

A recent prospective study has also compared peritoneal lactate, pyruvate, glucose and glycerol assessment with daily clinical scoring (leak scores and the Dutch Leakage Score system). This study showed that, in cases of AL, peritoneal lactate concentration increases over time and its assessment can have greater sensitivity, specificity and better PPV and NPV than clinical scoring systems. The median day for an AL diagnosis with a change in lactate ≥6.3 mM was 1.6, whereas for leak scores and for the Dutch Leakage score system, it was 3.3 and 7 days, respectively [154].

### 10.2. pH

To investigate pH as an ischaemic AL biomarker, one study has measured intestinal mucosal pH with tonometry. pH measurements were taken using a catheter placed at a colorectal anastomosis site through the anus. Imaging performed on the 6th day following surgery was used for symptomatic and asymptomatic AL diagnosis. Results indicated that in the first 24 h, mucosal pH values were significantly reduced in patients who subsequently developed an AL. Using a pH cut-off value of <7.28 in the first 24 h was associated with a 22-times greater risk of AL, with a sensitivity of 28% and specificity of 98% for AL prediction [155]. A further study measured peritoneal drain fluid pH in the first 12 days following colorectal surgery. Similar to the previous study, results indicated that pH values were significantly lower in patients which developed an AL that needed revision surgery. Using a cut-off pH value of <6.978 on the 3rd day following surgery had a sensitivity of 98.7% and specificity of 94.7% for predicting an AL. Interestingly, a decline in pH was seen in all patients preceding their AL diagnosis [156].

### 10.3. Tissue Oxygenation

The intra-operative use of Clark O_2_ electrodes, as previously described, has been investigated for their ability to predict AL. However, these studies overlooked their applications for post-operative use. The concept of using miniaturised Clark O_2_ sensors to provide post-operative intestinal ptO_2_ measurements has begun to be investigated by the Implantable Microsystems for Personalised Anti-Cancer Therapy (IMPACT) programme. Our group has developed novel implantable miniaturised Clark-type electrochemical O_2_ sensors [157] and methylene blue-based electrochemical and ion sensitive field-effect transistor (ISFET) pH sensors [158]. The idea that these sensors could be placed intra-operatively around the anastomotic site and be left in situ would allow clinicians to continuously monitor peri-anastomotic intestinal ptO_2_ and pH throughout the post-operative recovery period. This type of continuous monitoring system would help to identify patients at risk of developing an AL due to poor or deteriorating peri-anastomotic intestinal ptO_2_. It would also allow clinicians the ability to assess the efficacy of interventions designed to improve intestinal ptO_2_ and prevent a leak from occurring. The electrochemical O_2_ sensor has undergone initial *in vivo* validation in a rat model [159]. The results from this study showed that sensors, placed on intestinal serosal surfaces, were able to provide continuous, real-time ptO_2_ readings. These sensors also recognised and reported dynamic intestinal ptO_2_ changes that occurred with hypoxaemic and ischaemic challenges. The authors suggested that although further research is required, this pre-clinical study demonstrated the potential use of miniaturised implantable medical devices for intestinal surgery.

## 11. Biomarkers of Inflammation

A wide range of inflammatory mediators, such as acute-phase proteins, cytokines and growth factors, are released into the peritoneal cavity and bloodstream following abdominal surgery [160]. If these substances are to be used as part of a precision medicine approach in intestinal surgery, then studies have to show their ability to differentiate the normal physiological responses to surgery from clinically important complications such as AL.

### 11.1. C-Reactive Protein, Albumin and Procalcitonin

CRP, a hepatic acute-phase reactant with a half-life of 19 h, is typically found at low levels (0.8 mg/L) in the blood of healthy individuals. CRP levels can rise dramatically in response to inflammatory cytokines such as interleukin (IL)-6 (IL-6), tumour necrosis factor-α (TNF-α) and IL-1β. This can occur as part of an acute-phase inflammatory response due to infection, tissue damage and neoplasia [161]. Post-operative serum CRP levels are routinely assessed as part of standard care practices to provide information on clinically significant inflammation and post-operative complications. Unfortunately, its use in resection and anastomotic patients is still contentious, with studies demonstrating poor CRP specificity for AL diagnosis, with levels only becoming significantly raised when clinical symptoms become apparent [162,163,164].

In contrast to these results, recent research has shown that serum CRP levels can become elevated several days before clinical AL diagnosis and are significantly raised in comparison to patients who have an uneventful post-operative recovery [165,166,167,168,169,170,171,172,173,174,175,176,177]. Currently, the main issue with using serum CRP levels for AL prediction or diagnosis is the lack of definitive cut-off values. Cut-off values in these studies alone ranged from 123 to 245 mg/L, which were measured between 3 and 5 days following surgery. In a meta-analysis of seven clinical trials which included 2483 patients, results suggested that serum CRP cut-off values of 172 mg/L, 124 mg/L and 144 mg/L on the 3rd, 4th and 5th days following surgery possessed an NPV of 97% for excluding an AL [178]. Furthermore, in a recent prospective international study of 933 colorectal resection and anastomosis patients, of which 41 developed an AL, serum CRP levels were assessed pre-operatively and continued for 5 days post-surgery. Results indicated that a change in CRP levels >50 mg/L over any 2 post-operative days had a sensitivity of 85% for diagnosing an AL and an NPV of 99% for ruling it out. A change in CRP >50 mg/L between days 3 and 4 or 4 and 5 had an even higher specificity of 97%. The authors highlighted the value of CRP trajectory assessment for its ability to rule out an AL [179].

Albumin, also an acute phase protein, has been proposed as an indicator of surgical stress and can be used to predict the development of post-operative complications [180]. Hypoalbuminemia has also been suggested to be an AL risk factor for colorectal resections as part of treatment for ovarian cancer [181]. A novel indicator, the C-reactive protein:albumin ratio (CAR), has been used to identify patients at risk of post-operative complications after colorectal surgery [182]. This study showed that CAR measurement provided greater diagnostic accuracy than assessing CRP or albumin levels alone. In one recent retrospective study of 1068 elderly colorectal anastomotic patients, the AL predictive value of CAR was investigated. Using a pre-operative CAR cut-off value of 2.44, the assay had a sensitivity of 61% and specificity of 80% for predicting an AL. Surgical time and pre-operative CAR were also both identified as independent AL risk factors [183].

Procalcitonin (PCT), the prohormone of calcitonin, is produced by thyroid parafollicular C-cells. PCT is typically found in low levels (<0.05 ng/mL) in the blood of healthy individuals. Bacterial infections have been shown to induce PCT release from all differentiated cell types, which can occur within 2–3 h following infection and is related to the presence of bacterial endotoxins and inflammatory cytokines such as TNF and IL-6. Patients with serum PCT levels >2 ng/mL have been associated with bacterial infections, but levels >700 ng/mL can be seen in cases of severe sepsis [184]. Serum PCT levels, in contrast to CRP, do not become raised secondary to inflammation of a non-infectious origin and its use for early AL diagnosis has been investigated.

One study involving 157 colorectal resection and anastomotic patients demonstrated that serum PCT levels in the range of 1.4–4.62 ng/mL measured on the 1st day following surgery predicted those that subsequently developed an AL. These values were significantly higher than that seen in patients who recovered uneventfully (0.09–0.44 ng/mL). Using a PCT cut-off value of 1.09 ng/mL on the 1st day following surgery gave sensitivity and specificity values of 87% for AL prediction. The authors suggested that PCT could be used at this early post-operative time point to identify high-risk patients [185]. A similar study, again involving colorectal cancer resection and anastomosis patients, demonstrated that PCT measured on the 3rd day following surgery could identify patients at low risk of AL development. Using 3.83 ng/mL as a PCT cut-off value gave a sensitivity of 75% and specificity of 100% for AL prediction [168]. These results are supported by another study that also concluded that PCT levels measured over the first 5 days following surgery are a reliable predictor of AL after colorectal surgery. Using a PCT cut-off value of 0.31 ng/mL on the 5th day following surgery was shown to have 100% sensitivity, 72% specificity, 100% NPV and 17% PPV for AL. The authors suggested that patients with elevated serum PCT levels on post-operative days 3–5 warranted further assessment before discharge [186]. These results have been supported in other studies [166,170].

Many of these studies assessed both CRP and PCT levels simultaneously and it has been proposed that measuring both can improve AL diagnosis. In the recent PREDICS study involving 504 colorectal resection and anastomosis patients, the study demonstrated that a PCT cut-off value of 2.7 ng/mL had an NPV of 96.9% and specificity of 91.7% on the 3rd day following surgery, whereas a cut-off value of 2.3 ng/mL on day 5 had an NPV of 98.3% and specificity of 93% for AL diagnosis. CRP also exhibited good NPV 96.4% on the 3rd day (cut-off value 169 mg/L) and 98.4% on the 5th day (cut-off value 125 mg/L). Combined CRP and PCT assessment further improved AL diagnosis [170]. These results have been supported by a more recent study that suggested that CRP and PCT levels were higher on post-operative days 1–3 in patients who subsequently developed an AL. The authors suggested that these markers could be used to allow early patient discharge in those with low risk of developing an AL [187].

### 11.2. Cytokines, Tumour Necrosis Factor-α and Growth Factors

Cytokines such as IL-1, IL-6, IL-10 and TNF-α are polypeptides with known roles in immune responses. In response to surgical trauma, they regulate physiological responses and induce the production of hepatic acute-phase proteins, whilst, in response to sepsis, they can mediate systemic inflammatory responses [188,189,190]. Raised IL-1b, IL-6 and TNF-α levels have also been associated with surgical stress, including lengthier operating times, haemorrhage and high peritoneal bacterial counts [191,192,193]. Within the first few hours following abdominal surgery, these substances are released from the surgical site. During this period, studies have shown that peritoneal cytokine levels are raised to a greater degree than serum levels. This provides evidence, similar to the ischaemic biomarkers, that peritoneal rather than serum biomarker levels are more representative of localised tissue changes [194,195]. In patients that have uncomplicated post-operative recoveries, peritoneal cytokine levels typically decrease within 24 h following surgery [195]. However, cytokine dynamics that occur with an AL follow a significantly different course.

Raised peritoneal levels of IL-6 and TNF-α have been shown in numerous studies to occur as early as the 1st day following surgery in patients who go on to develop an AL [148,196,197,198,199]. Further studies, however, have demonstrated that their levels only become elevated from the 3rd post-operative day [200,201]. An important observation from all these studies was that, in AL patients, IL-6 and TNF-α levels for the first 5 post-operative days remained elevated, whereas, in patients that recovered uneventfully, their levels remained low or even decreased. Although a further study observed no differences between a control group and patients who developed an AL in their IL-6 and TNF-α levels over the first 7 days following surgery, the results demonstrated that TNF-α levels rapidly rose 24 h before a surgical diagnosis of AL was made [202]. A more recent case–control study investigated serum and peritoneal IL-6 levels on the 2nd and 4th days following colorectal surgery. In total, 30 AL and intra-abdominal abscesses (infection group) were compared with 30 uneventful recovery patients (control group). These results demonstrated that higher peritoneal levels in the infection group were seen on the 2nd and 4th days, whereas serum levels only became significantly elevated on the 4th day [203]. A further study identified that serum IL-6 levels on the 3rd post-operative day were significantly elevated in AL patients with similar sensitivity to that of CRP. Interestingly, the relative change in pre-operative to post-operative IL-6 levels was significantly higher in AL patients, with granulocyte-colony stimulating factor also showing similar changes [204]. Increased peritoneal levels of IL-1, IL-10, vascular endothelial growth factor, epidermal growth factor and platelet-derived growth factor have also been suggested to occur in patients who develop an AL and sepsis following colorectal surgery [148,193,196,197,198,201,203].

### 11.3. Leukocytes, Neutrophils and Intestinal Damage Markers

White blood cells (WBC) play a crucial role in wound healing through microorganism elimination. It had been proposed that the WBC count (WBCC) can reflect the extent of inflammation within the body or surgical site. As an AL creates significant inflammatory responses, several studies have investigated whether assessing leukocyte and/or neutrophil numbers in blood can aid AL diagnosis.

In one retrospective study of 1187 colorectal cancer patients, CRP levels and WBCC were assessed for the first 5 days following surgery. CRP levels, in line with other studies, measured on the 4th day provided the highest diagnostic accuracy for identifying post-operative complications, whereas WBCC contributed little information [174]. These results were supported by other retrospective [176] and prospective studies [170]. A further study demonstrated that in patients who developed an AL, increased WBCC only occurred after 6 days following surgery [165]. In a smaller study of 129 laparoscopic colorectal surgery patients, systemic CRP levels and WBCC were assessed. Using a CRP cut-off value of >200 mg/L on the 3rd day following surgery had a sensitivity of 68% and a specificity of 74% for predicting septic complications, whilst using a WBCC cut-off value of >12 × 10^9^ on the 2nd day had a sensitivity of 90% and a specificity of 62%. The authors concluded that systemic CRP levels and WBCC were poor early diagnostic markers for predicting post-operative septic complications (including AL) [164]. Assessing neutrophil:lymphocyte ratios (NLR) has also been described as a method for AL prediction. One study demonstrated that NLR on the 4th day following surgery had prognostic value, with higher NLR identified in AL patients. An NLR cut-off value of 6.5 had a sensitivity of 69%, specificity of 78%, PPV of 49% and NPV of 88% for AL diagnosis. NLR were also significantly higher at this time point in patients who subsequently died in the post-operative period [171].

Calprotectin makes up ~60% of the cytosolic proteins found within neutrophils and is a recognised marker of neutrophil activation [205]. Studies have begun to investigate calprotectin as an inflammatory biomarker for early AL diagnosis. In one retrospective study of 84 colorectal cancer patients, serum CRP and calprotectin levels were assessed for 5 days following surgery. In patients that developed an AL, calprotectin levels became significantly elevated on the 2nd day following surgery (588 ng/mL) compared to those that went on to recover uneventfully (366 ng/mL). Calprotectin levels in AL patients also remained elevated throughout the 5 days. Although the authors suggested that calprotectin levels could be used to diagnose an AL, improved diagnostic accuracy was obtained when combined calprotectin and CRP assessment was performed on the 3rd day following surgery. This assay provided a sensitivity of 100%, specificity of 89%, positive likelihood ratio of 9.09 and negative likelihood ratio of 0.00 [167]. Similar results have been seen in a further study which identified raised pre-operative and post-operative calprotectin levels on the 1st, 3rd and 5th days following surgery in patients which developed an AL, whereas CRP levels only became elevated on the 3rd and 5th days, with no WBCC changes being observed. The authors again suggested that combined calprotectin and CRP assessment might aid early AL diagnosis [206].

Faecal calprotectin has also been used for assessing inflammation secondary to colorectal cancer and inflammatory bowel disease and its role in predicting AL has been investigated. In one study of 100 colorectal anastomotic patients, in which 11 developed an AL, faecal calprotectin levels were assessed 4 days after surgery. Results indicated that faecal calprotectin was significantly higher (>300 μg/g) in patients who developed an AL compared with those that recovered uneventfully (<90 μg/g). Faecal calprotectin levels assessed in combination with CRP using a cut-off value of 120 mg/L provided a sensitivity of 85%, specificity of 95% and an NPV of 95% for AL diagnosis [207].

Plasma markers of intestinal damage, such as liver, ileal bile acid and intestinal fatty acid-binding proteins, have also been investigated as predictive AL biomarkers. Using a pre-clinical intestinal resection and anastomosis rat model, post-operative serum intestinal fatty acid-binding protein level was shown to be raised as early as the 3rd day following surgery in those that developed an AL [208]. One human study demonstrated that pre-operative intestinal fatty acid-binding protein levels >882 pg/mL had a sensitivity of 50% and specificity of 100% for predicting AL [167]. A further study demonstrated that urinary intestinal fatty acid-binding protein and the urinary intestinal fatty acid-binding protein:creatinine ratio on the 3rd day following colorectal surgery were significantly elevated in patients with an AL. The authors suggested that this urine-based assay could be used as a non-invasive assay for AL diagnosis [209].

### 11.4. Macrophage Biomarkers

Produced by macrophages, lysozyme is a substance which disrupts the cell wall of Gram-negative bacteria. As lysozyme plays an important role in the inflammatory response to sepsis and trauma, studies have begun investigating whether it could be used as an AL biomarker. One study demonstrated that peritoneal lysozyme levels in patients on the 1st day following a low anterior resection who had an uneventful post-operative recovery were 5.5 mg/L. Significantly higher levels were seen in patients with clinical (180 mg/L) and radiological (153 mg/L) evidence of AL [210]. Although the authors suggested that lysozyme could be used for early AL diagnosis, the electrophoretic technique used had significant practical restraints in terms of its usefulness as a rapid AL diagnostic test as the gel required overnight soaking.

Neopterin, also produced by macrophages, is recognised as a biomarker of T helper cell activation and plays a significant role in mediating inflammatory responses. Neopterin production is stimulated by interferon-γ and can be detected in urine, cerebrospinal fluid and blood. Increased neopterin levels have been associated with viral, bacterial and parasitic infections, autoimmune diseases, cancer [211], sepsis [212] and multiple organ dysfunction syndrome [213]. In terms of AL, one study has investigated pre- and post-operative blood, urine and peritoneal fluid neopterin levels in colorectal resection and anastomosis patients [214]. Results demonstrated that the pre-operative urinary neopterin:creatinine ratio was significantly higher in patients that went on to develop an AL compared to those that recovered uneventfully (139.5 µmol/mol vs. 114.8 µmol/mol). Patients that developed complications also had higher urinary and peritoneal neopterin levels following surgery. The authors suggested that high pre-operative levels of urinary neopterin could identify AL high-risk patients and that monitoring post-operative urinary and peritoneal fluid neopterin levels could be useful for early AL diagnosis.

### 11.5. Hyponatraemia

Hyponatremia, although a commonly diagnosed electrolyte disorder, has been proposed as an inflammatory biomarker and a potential indicator of peritonitis [215]. Sodium levels are predominantly maintained via osmotic vasopressin release mediated by intra-vascular volume. However, research has shown potential immune-neuroendocrine pathways involving IL-6 which may have a role in non-osmotic driven vasopressin release in response to inflammation [216,217]. Hyponatremia (<136 mmol/l) and leukocytosis (>10 × 10^9^/l) have subsequently been investigated as predictive AL biomarkers following colorectal surgery [218]. Results from this study of 1025 patients identified that 23% (*n* = 19) of AL patients and 15% (*n* = 69) of patients who recovered uneventfully had hyponatremia. Leukocytosis was identified in 12 of the 19 patients with hyponatremia and an AL. Hyponatraemia on the 5th day following surgery had a sensitivity of 23%, specificity of 93%, NPV 97% and PPV of 5% for AL diagnosis. The combined presence of hyponatremia and leukocytosis had a sensitivity of 68%, specificity of 75%, PPV of 18% and NPV of 97%. The authors suggested that, due to low sensitivity (23%), hyponatremia absence cannot exclude the presence of an AL. However, as the specificity of hyponatremia for an AL was high, especially when it occurred in the presence of leucocytosis, this result should raise suspicion of an AL being present. Further prospective trials are needed to confirm these results.

## 12. Biomarkers of Tissue Repair

MMPs are a group of zinc-dependent endopeptidases that are involved with extracellular matrix (ECM) remodelling. Secreted as an inactive pro-enzyme, they become active following proteolytic cleavage [219]. Physiological and pathological processes involving tissue repair and regeneration depend on the balance between MMP proteolysis and its prevention by tissue inhibitors of metalloproteinase (TIMP) [220]. MMPs have been suggested to play a role in AL development through inhibition of collagen synthesis. Although collagen type I and III genes are normally overexpressed at anastomotic sites, in a rat model, maximal gene expression was not reached until 7 days following surgery [221]. Further animal models have demonstrated that colonic peri-anastomotic healing (as shown by higher bursting pressures, improved structural layers and increased collagen production) was improved through MMP inhibition [222,223,224,225]. Furthermore, human colonic tissue from patients with poor anastomotic healing has demonstrated higher mucosal MMP-1 and MMP-2 expression and higher submucosal MMP-2 and MMP-9 expression. Interestingly, colonic samples from AL sites demonstrated a significantly lower collagen type I:III ratio compared to uncomplicated anastomotic sites [226].

In a study of 58 colorectal anastomotic patients, peritoneal levels of MMP-1, 2, 3, 8 and 9 and TIMP-1 and 2 were assessed for 8 days following surgery. Differential levels of MMP and TIMP were assessed on each day along with total MMP activity. Their levels were shown to vary depending on the operation type and duration, amount of haemorrhage and with the occurrence of post-operative complications. Only MMP-2 and MMP-9 levels positively correlated with the development of post-operative complications, whereas TIMP-1 and TIMP-2 levels demonstrated a negative correlation. The authors suggested that peritoneal MMP and TIMP may act as biomarkers of intestinal wound healing and surgical outcome. However, as the patient cohort within the study was heterogeneous and because the types of post-operative complications were not specified, further studies are required [227]. In contrast to these results, a pilot study of 29 low anterior resection patients had their peritoneal fluid levels of MMP-1, 2, 3, 7, 8, 9 and 13 assessed every 4 h following surgery. Only MMP-8 and 9 were significantly increased in the 10 patients who developed an AL compared with the 19 patients who had an uneventful post-operative recovery [228].

In a recent systematic review, which included animal and human studies, the role of tissue, blood and peritoneal MMP levels in the development of AL was investigated. The results from 12 studies suggested that elevated MMP-9 levels were the most consistent finding in patients that developed an AL [229]. The authors claimed that although these studies suggested that tissue or peritoneal fluid levels of MMP and/or TIMP could act as biomarkers for AL, the number of studies and number of patients used were small. In addition, the inconsistent results for specific MMPs suggests that further investigations are required.

## 13. The Intestinal Microbiome and Bacterial Contamination

The intestinal microbiome has been shown to play a role in the development of AL and can be influenced by multiple peri-operative factors [71]. During intestinal surgery, inadvertent spillage of intestinal contents can cause bacterial contamination of the abdominal/pelvic cavities. In the majority of patients, immune responses deal with this contamination and their post-operative recovery is not compromised. However, in patients that develop anastomotic dehiscence, irrespective of its cause, significant and ongoing bacterial contamination can overwhelm the patient’s immune system. A 5-year prospective trial of patients diagnosed with abdominal sepsis syndrome (inflammatory response with organ failure secondary to digestive tract perforation) identified multiple micro-organisms to be present within their abdominal fluid. The peritoneal microbial flora composition of these critically ill patients also varied depending on site of the intestinal perforation. Following a colorectal perforation ~70% of intra-operative fluid samples contained aerobic Gram-negative bacteria (*Escherichia coli, Klebsiella* and *Pseudomonas* species) whilst the predominant anaerobic species was *Bacteroides*. Gram-positive bacteria (*Enterococci* and *Staphylococci*) were found in ~50% of colorectal perforation cases. Antibiotic treatment was also shown to change the microbial flora, causing Gram-negative bacterial counts to drop whilst Gram-positive bacterial counts increased [230]. These results are supported by another study which identified similar peritoneal microbial flora constituents following intestinal perforation [231].

### Bacterial Load

Assessment of peritoneal bacterial contamination has been investigated as an early AL diagnostic biomarker. One study obtained post-operative peritoneal fluid samples for microbial culture from 56 low anterior resection patients. In eight patients that had an AL confirmed by imaging, *Escherichia coli*, *Bacteroides, Klebsiella* and *Pseudomonas* species were identified on the 1st, 3rd and 5th days following surgery. Unfortunately, the specificity of using culture results as an AL diagnostic test was low as several false positive cases occurred in which all four bacterial species were identified in a patient without an AL [200]. The clinical applicability and usefulness of peritoneal microbial cultures for rapid AL diagnosis is also severely limited by the time required to grow laboratory cultures. To overcome this issue, studies have investigated other techniques in which bacteria or bacterial components can be identified.

In a pilot study of 17 colorectal anastomotic patients, a reverse transcription-polymerase chain reaction (RT-PCR) assay designed to identify *Escherichia coli* and *Enterococcus faecalis* was performed on 10 culture-positive and 7 culture-negative peritoneal fluid samples. While the RT-PCR results agreed with microbiological culture results, the assay suffered from a lack of specificity, with four false positive results being identified. Although these false positives all resulted from samples originating from a single patient with a surgical site infection, the authors suggested that RT-PCR may be too sensitive for AL diagnosis, leading to over-diagnosis and over-treatment [232]. To further investigate this, the authors used the same *Escherichia coli* and *Enterococcus faecalis* RT-PCR assay in a multicentre study involving 243 left-sided colonic anastomotic patients. In the 19 patients that developed a symptomatic AL, *Escherichia coli* concentration was significantly increased on the 4th and 5th days following surgery, whereas *Enterococcus faecalis* was significantly increased on days 2, 3 and 4. The authors suggested that *Enterococcus faecalis* on the 3rd day had the highest diagnostic accuracy, with a sensitivity of 92.9% and NPV of 98.7% of AL diagnosis. Although a number of false positives were still identified, the authors further suggested that the absence of *Enterococcus faecalis* on day 3 could potentially exclude the presence of an AL [233].

The use of online infrared absorption to spectroscopically detect bacteria in peritoneal fluid samples has also been investigated as a means of identifying bacterial contamination. To provide proof-of-principle, one study demonstrated that this technique could differentiate between peritoneal fluid samples obtained from a patient who recovered uneventfully from those of a patient who developed post-operative complications with highly contaminated peritoneal fluid. A significant increase in infrared absorption occurred as contamination levels increased. The authors suggested that although the technique cannot provide information on the source of the contamination, it has the potential to be used as a bedside AL early-warning system [234]. Further studies are required to assess the use of optical systems as AL diagnostic tools. 

Peritoneal levels of endotoxins and lipopolysaccharide (LPS), which forms part of the cell wall of Gram-negative bacteria (including intestinal commensals), have been suggested as diagnostic biomarkers of peritonitis and AL [235]. One study measuring peritoneal LPS levels from 22 colonic resection patients demonstrated significantly raised LPS levels on the 1st and 3rd days following surgery in three patients that developed an AL. Although LPS differences between patients that recovered uneventfully and those that developed an AL were great, standard deviations between patient groups were large. Two patients also had surgery for perforated sigmoid diverticulitis, so elevated LPS levels may have been due to pre-existing bacterial contamination [236]. Currently, LPS is not routinely measured in clinical laboratories and further studies are required before its usability as an AL biomarker can be determined.

## 14. Limitations of Biomarkers of Anastomotic Leakage and Future Perspectives

Biomarkers that can monitor intestinal healing, identify patients at high risk of developing an AL or provide early AL diagnosis, have the potential to significantly change how we manage resection and anastomosis patients. Although pre-clinical and clinical research continues to identify novel biomarkers for these purposes, none have made it into clinical use. Stumbling blocks for the translation of study results into practice changing policies is complicated but can be related to study design and the usability of the assay itself.

Direct comparison between biomarker studies is difficult, not only because many use heterogeneous patient populations, but also because of a lack of a single, clearly defined AL definition. Some studies use asymptomatic patients with diagnosis based on imaging, whilst others only use patients exhibiting clinical signs. These clinical signs can also be wide-ranging, from non-specific to the presence of a faecal fistula or multi-organ failure. A significant number of studies are also retrospective in nature. Although this means large sample sizes can be obtained, studies can run for several years, over which time the surgical team, surgical techniques and post-operative management practices can change significantly. Studies also differ in the timings of blood tests and/or peritoneal fluid analysis, with biomarker levels rarely evaluated specifically in terms of anastomotic position (colonic and rectal resections) or the underlying disease process. They also fail to account for medications or treatments that may alter inflammatory responses, such as statins, steroids, chemotherapy or radiotherapy. All these considerations are especially important when AL cut-off values are determined from study results; these differences will undoubtably have contributed to the significant variations in AL cut-off values reported across these studies. Standardised, multicentre prospective studies are needed to overcome these issues.

A large number of studies suggest that peritoneal fluid samples obtained from abdominal drains provide a better indication of the peri-anastamotic tissue environment than blood samples. Although this may be true, studies typically fail to document drain location and type, which makes comparing results from different studies challenging. It has also been shown that drain location can influence drain fluid composition [237]. As gross body movements, including coughing, can affect drain location, this means that changes in drain fluid biomarker levels may be solely due to changes in drain location rather than fluctuations in patient status or intestinal healing. The clinical value of using peritoneal drains after a resection and anastomosis also remains a contentious issue. Several studies and meta-analyses have not shown any benefit of peritoneal drainage in decreasing AL incidence [238,239,240]. If surgeons are unwilling to routinely place them at surgery, then basing a biomarker assay on drain fluid will ultimately fail to reach clinical use. Strong clinical evidence proving that peritoneal drain fluid analysis is useful for the management of these patients is needed to allow peritoneal drainage to become routine and no longer controversial.

In terms of developing a clinically usable assay, certain biomarkers have inherent problems. Biomarkers such as cytokines and MMP are labile, meaning that peritoneal fluid analysis has to be performed immediately. Expensive and labour-intensive assays such as enzyme-linked immunosorbent assays (ELISA) or PCR technologies also have clinical limitations as laboratory processing, even if available in the hospital, incurs inherent time delays in reporting results. Studies that have investigated bacterial contamination have also shown a lack of clinical usability, either through time delays associated with growing cultures or through high false positive rates with RT-PCR assays.

**Figure 2:**
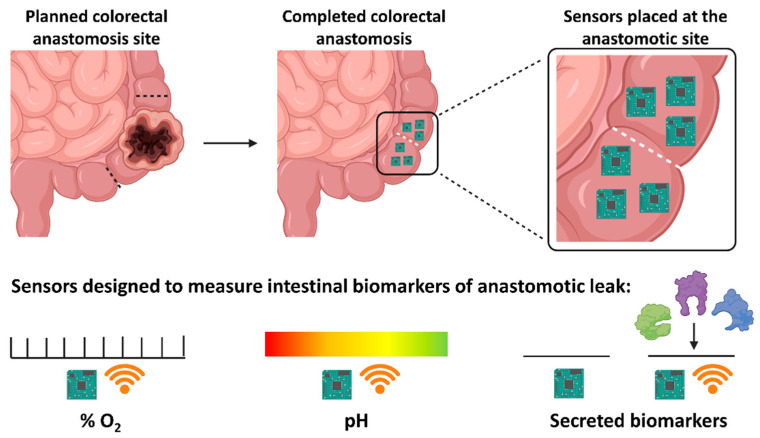
Future applications of advanced technologies for measuring anastomotic leak biomarkers. Implantable sensors placed intra-operatively around the anastomotic site could be left in situ throughout the post-operative recovery period. This concept would allow clinicians to continuously monitor peri-anastomotic biomarkers such as O_2_, pH, C-reactive protein, lactate and pyruvate levels. This type of continuous monitoring system would help to identify patients at risk of developing an AL due to poor or deteriorating peri-anastomotic intestinal ptO_2_. It would also allow clinicians the ability to assess the efficacy of interventions designed to improve intestinal ptO_2_ and prevent a leak from occurring. Figure created in Biorender.

Researchers are continuing to investigate methods to overcome these issues. Multidisciplinary projects involving engineers, chemists and clinicians are looking at ways in which implantable medical devices and sensor technologies could be utilised for such purposes. Studies such as the IMPACT project have already provided initial results regarding the development of miniaturised O_2_ and pH sensors. Further research will undoubtably lead into the creation of sensors for the detection of the most promising AL biomarkers, such as CRP, lactate and pyruvate (Figure 2). Wireless technology also creates the possibility of producing a biodegradable implant, which could be fixed around the anastomosis to remotely provide information about the tissue environment. Although this research is still in its infancy, technological advancements may ultimately deliver a simple, acceptable and low-cost method of measuring known AL biomarkers from peritoneal fluid directly surrounding an anastomosis or from peri-anastamotic serosal surfaces on which the sensors are placed. 

## 15. Conclusions

In the field of colorectal cancer research, significant advancements have been made in the identification of diagnostic and predictive biomarkers of AL. This research is driven by the clinical need to identify patients at high risk of developing an AL and to diagnose AL earlier than current protocols allow. The ideal biomarker would allow for rapid, cost-effective and reliable prediction or detection of an AL in a time frame that allows clinicians to instigate interventions that minimise patient morbidity and mortality. Here, we have highlighted the current, most promising potential candidate biomarkers, including ischaemic metabolites, inflammatory markers and bacterial components. Although none of these biomarkers have yet been validated in large-scale clinical trials, with none in routine clinical use, ongoing biomarker research in the field of intestinal surgery holds much promise. The incorporation of such biomarkers outlined in our review with other techniques, such as clinical status, routine haematological and biochemical analysis and imaging, has the potential to deliver an overall precision medicine package that could significantly enhance the effectiveness of a patient’s post-operative care. There is a need, now more than ever, to utilise our knowledge of these biomarkers in carefully designed prospective, multicentre studies. These trials should be designed to investigate whether proactive post-operative patient management based on predictive biomarker levels can be used to reduce AL rates. There is confidence within the scientific community that precision medicine, through the incorporation of biomarker analysis, will finally be realised for intestinal resection and anastomosis patients in the decades to come.

## Figures and Tables

**Table 1:** Risk factors associated with the development of an anastomotic leak.

Patient Factors		Surgical Factors	
Age Malnutrition Steroid use Diabetes Hypertension Tobacco use	Cardiovascular disease Gender Alcohol use ASA fitness score Diverticulitis Leukocytosis	Poor anastomotic blood supply Concurrent surgical procedures Poor colonic preparation Peri-operative blood transfusion Anastomotic ischaemia or tension Emergency resection	Intra-operative sepsis Peritonitis Operative time >3 h Pre-operative radiotherapy Anastomotic location Bowel obstruction

**Table 2:** Peri-operative techniques for AL risk prediction and diagnosis.

Pre-Operative	Intra-Operative	Post-Operative
Surgical factors Patient factors Predictive scoring systems Blood samples Urine samples	Tissue appearance Air leak test Endoscopy Intestinal tissue perfusion Intestinal tissue oxygenation	Scoring systems Clinical assessment Routine bloodwork Imaging Biomarkers: ischaemic, inflammatory, bacterial

## Data Availability

Not applicable.
